# Metastatic Rectal Adenocarcinoma in Familial Adenomatous Polyposis: A Case of Socioeconomic Factors Influencing Surveillance Endoscopy

**DOI:** 10.7759/cureus.28591

**Published:** 2022-08-30

**Authors:** Paul Guzik, Harold J Duarte, Nour A Parsa

**Affiliations:** 1 Gastroenterology and Hepatology, Loma Linda University Medical Center, Loma Linda, USA; 2 Internal Medicine, Kettering Medical Center, Kettering, USA

**Keywords:** familial adenomatous polyposis, colorectal cancer, ileal pouch-anal anastomosis, socioeconomic factors, colon and rectal surgery, rectal cuff, early-onset colorectal cancer, pouchoscopy, colon cancer surveillance, fap

## Abstract

Familial adenomatous polyposis (FAP) is a rare syndrome caused by adenomatous polyposis coli (*APC*) gene mutation resulting in the development of hundreds of adenomatous colorectal polyps. The disease process usually manifests fully by the second decade of life. Total colectomy or restorative proctocolectomy is often required to prevent the development of colorectal adenocarcinoma. Routine surveillance following surgery is critical for the early detection of polyps or malignancy. We present a rare case of a 31-year-old male with a history of FAP status post total proctocolectomy with ileal pouch-anal anastomosis (IPAA) who presented with acute exacerbation of lower back pain and new-onset lower extremity paresthesia. Imaging demonstrated an aggressive T12 vertebral body lesion. Pathology following laminectomy demonstrated metastatic adenocarcinoma. Subsequent pouchoscopy revealed a distal 1.5-cm pedunculated lesion arising from remnant rectal tissue with pathology confirming moderately differentiated rectal adenocarcinoma. This patient underwent a prophylactic proctocolectomy 20 years prior to this admission but was lost to follow-up prior to any endoscopic evaluations. Despite postoperative surveillance guidelines and patient counseling, follow-up and recommended endoscopic evaluation are often inadequate. This case examines potential socioeconomic factors influencing the completion of surveillance endoscopy and also represents an opportunity to incorporate education and provide resources to patients with FAP to improve surveillance examinations and mitigate the development of preventable malignancies.

## Introduction

Familial adenomatous polyposis (FAP) is a rare autosomal dominant syndrome resulting in the development of hundreds of adenomatous colorectal polyps often during the second decade of life [1]. FAP results from a germ-line mutation in the adenomatous polyposis coli (*APC*) gene, a tumor suppressor gene also involved in about 60% of sporadic colorectal carcinomas (CRCs) and adenomas [2,3]. Although it is usually hereditary, nearly 30% of FAP cases are caused by de novo APC mutations [3].

Patients with FAP develop CRC at an average age of 39 years without surgery [4]. Surgery can drastically reduce, but not eliminate, the risk for CRC [5]. The most common surgical management involves either total colectomy with ileorectal anastomosis (IRA), restorative proctocolectomy with ileal pouch-anal anastomosis (IPAA), or proctocolectomy with ileostomy [6,7]. Restorative proctocolectomy with IPAA has been associated with worse functional outcomes (increased bowel frequency, night defecation, and use of incontinence pads) and complications (increased perianal irritation, anastomotic strictures, and need for reoperation within 30 days) compared to IRA but does have reduced malignant potential since nearly all colorectal tissue is removed [6,8-10]. Typically, a rectal cuff will remain in place; however, there have been exceptions to this with cuffless IPAA in the appropriate setting [11]. The cuffless IPAA differentiates itself by creating a direct anastomosis between the ileum and the anal tissue, completely removing the rectal cuff used in traditional IPAA. Postoperative surveillance is still required following all surgical approaches due to the risk for adenoma formation in the ileum, anal transition zone, or residual rectal tissue [6,8,12-15]. Pouchoscopy or ileoscopy is recommended at one- to two-year intervals indefinitely in those with a history of IPAA, and flexible sigmoidoscopy is recommended at six- to 12-month intervals indefinitely in those with a history of IRA [16].

We present a rare case of a young male with FAP presenting 20 years after restorative proctocolectomy with traditional IPAA, without interval surveillance examinations due to socioeconomic factors, now found to have metastatic adenocarcinoma arising from residual rectal mucosa (rectal cuff).

## Case presentation

**Figure 1:**
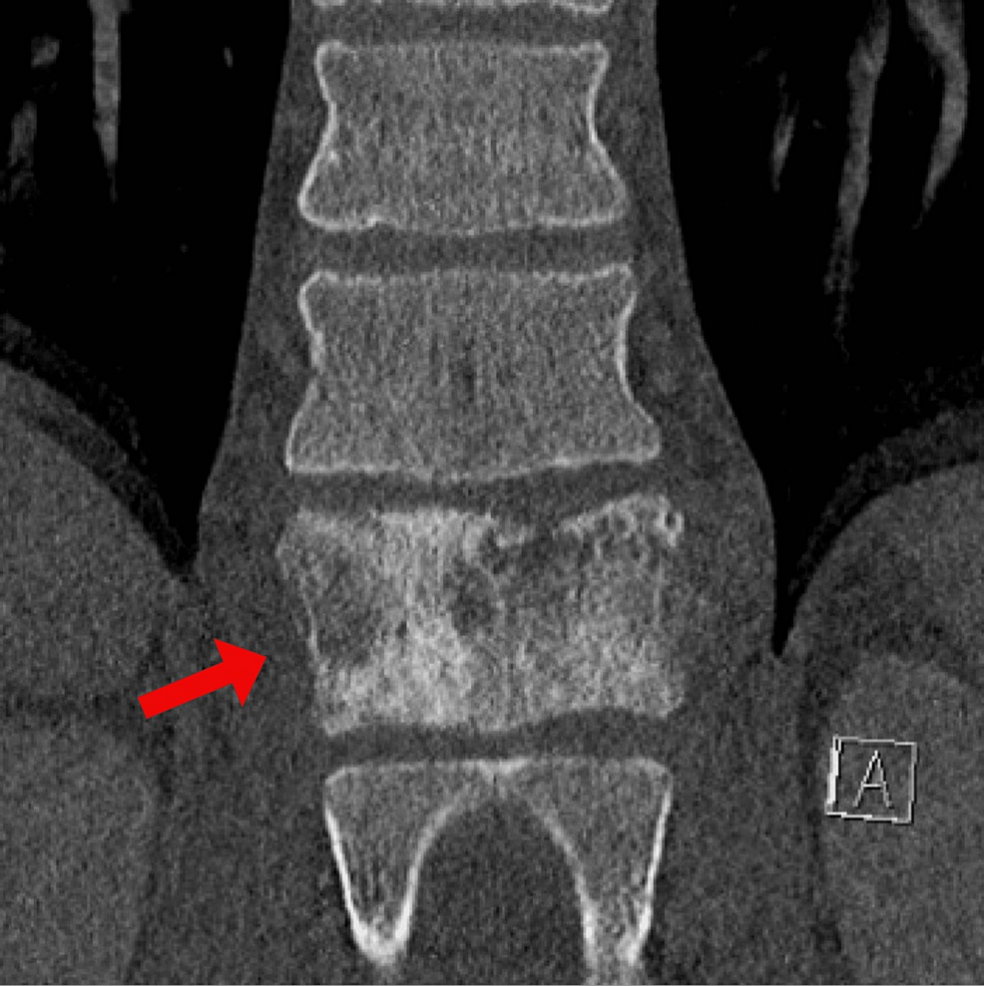
Heterogeneous, mixed sclerotic, and permeative T12 vertebral body lesion (red arrow) with paraspinal and significant epidural/foraminal soft tissue extension, resulting in severe central spinal stenosis.

**Figure 2:**
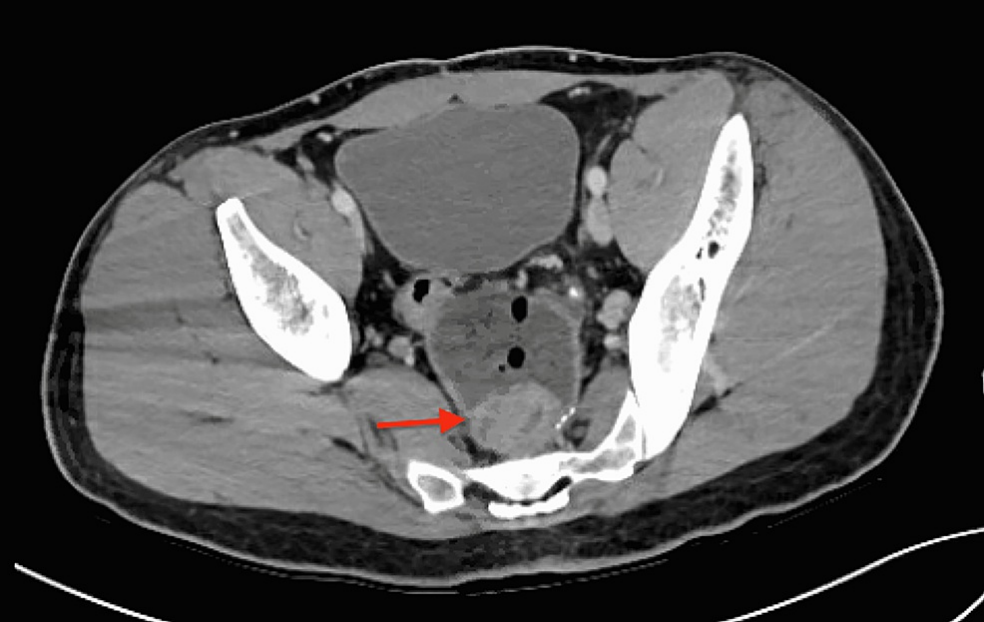
CT of the abdomen/pelvis with contrast demonstrating 2.7-cm rectal mass (red arrow). CT: computed tomography

A 31-year-old male with a history of FAP status post restorative proctocolectomy with IPAA presented following a mechanical fall related to one month of intermittent bilateral lower extremity paresthesia and four months of worsening back pain. Other than chronic diarrhea present since proctocolectomy, the patient did not endorse any recent gastrointestinal (GI) symptoms such as tenesmus, melena, hematochezia, or rectal bleeding. Computed tomography (CT) of the thoracic spine with intravenous (IV) contrast demonstrated an aggressive T12 vertebral body lesion with severe central spinal stenosis (Figure 1). CT of the chest, abdomen, and pelvis with IV contrast revealed postsurgical changes related to the rectum with an adjacent prominent 2.7-cm soft tissue mass and a notable 7-mm left pelvic sidewall lymph node (Figure 2). Additionally, a hypodense right hepatic lobe lesion measuring 1.8 × 1.5 cm was seen. Surgical pathology following laminectomy demonstrated metastatic adenocarcinoma with focal mucinous features and extensive necrosis, favoring lower gastrointestinal primary malignancy.

Upon further review, the patient was diagnosed with FAP at age 11 after recurrent episodes of abdominal pain and bloody stools. Colonoscopy at that time revealed extensive colonic polyposis with low-grade dysplasia on pathology. He subsequently underwent restorative proctocolectomy with traditional IPAA at age 12 with a genetic evaluation confirming *APC* gene mutation. Postoperatively, the patient did not follow up with a gastroenterology or surgery clinic, resulting in nearly 20 years without endoscopic surveillance examinations. His lack of surveillance and follow-up resulted from several factors but chiefly stemmed from the passing of his mother shortly after his proctocolectomy and lack of personal education and awareness of his condition. He also had several gaps in medical insurance coverage.

**Figure 3:**
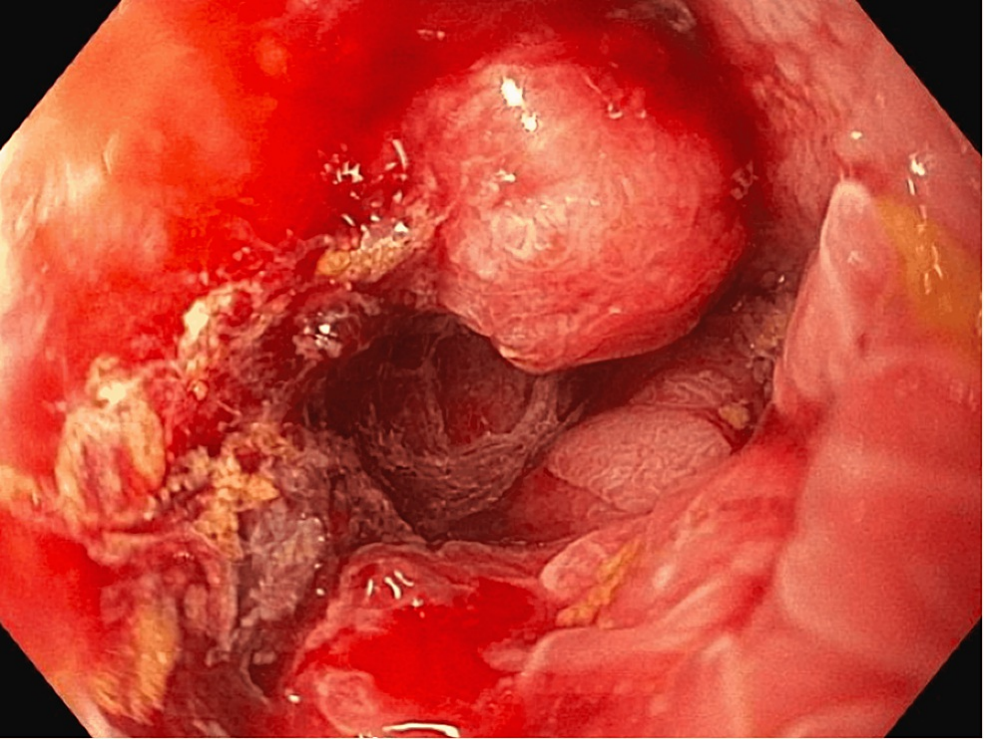
Broad-based 1.5-cm pedunculated lesion seen distally containing rectal tissue.

**Figure 4:**
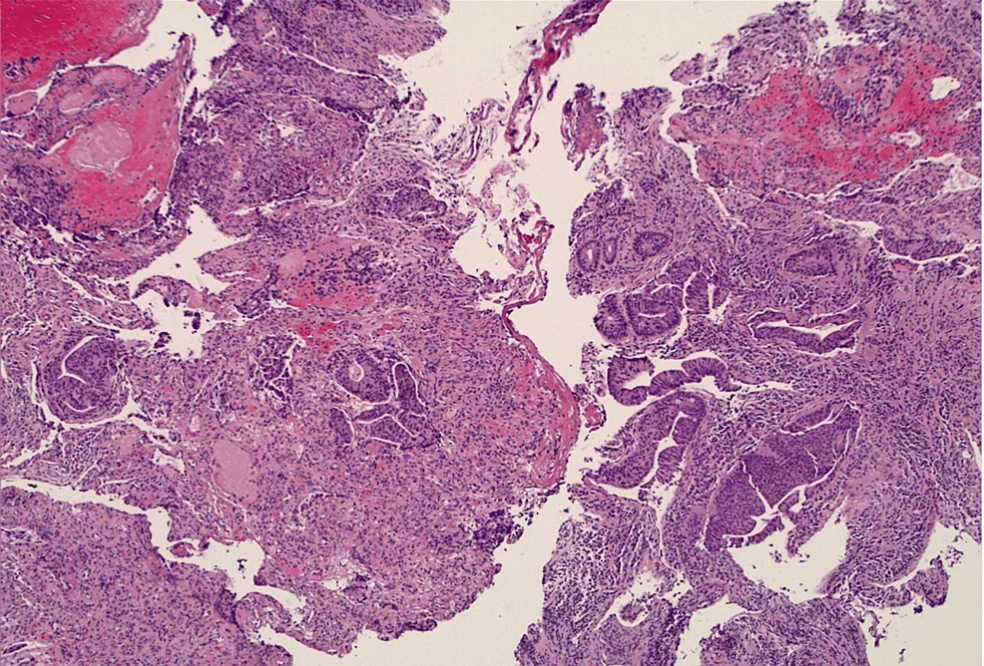
Infiltrative moderately differentiated adenocarcinoma. Microfoci highly suspicious for lymphovascular invasion.

An esophagogastroduodenoscopy (EGD) during this admission revealed a large periampullary polyp (Paris O-IIb) with pathology findings consistent with tubular adenoma. Pouchoscopy revealed a distal 1.5-cm pedunculated rectal lesion (Figure 3) with pathology confirming a moderately differentiated rectal adenocarcinoma (Figure 4). The patient started radiation therapy directed at the T12 vertebral body lesion while hospitalized, with plans for outpatient radiation oncology follow-up. Following hospital discharge, a positron emission tomography (PET) scan revealed F-fluorodeoxyglucose (FDG) foci near the rectal anastomosis, pelvic side wall lymph nodes, and both peripheral right hepatic lobe and T12 with epidural extension concerning for metastasis, as well as round bilateral chest wall and axillary lymph nodes with mild FDG activity, which was suspicious for malignancy. The patient was evaluated by medical oncology for initiation of systemic chemotherapy. Surgical oncology noted that the decision regarding surgery would be deferred until assessing the patient’s response to neoadjuvant chemotherapy.

## Discussion

The initial management of FAP focuses on reducing the high risk of malignant transformation in untreated patients, thereby decreasing the associated morbidity and mortality. If the polyp burden is manageable, colonoscopy can be performed every 1-2 years following the diagnosis [16,17]. Surgery is indicated in patients with >20 rectal polyps, large (>1 cm) polyps, and polyps with advanced histology or by patient preference [15]. Following surgery, surveillance endoscopy is critical for the early detection of polyps and malignancies. Even with restorative proctocolectomy, small residual rectal mucosal islands can remain and thus confer risk for malignant transformation as seen in this patient. Socioeconomic factors should also be considered as they may influence decisions regarding the optimal surgical approach. In this case, a cuffless IPAA may have been considered if the lack of future surveillance endoscopy was anticipated. Although postoperative surveillance is still recommended following cuffless IPAA, the malignant potential is theoretically reduced as all rectal mucosa is attempted to be removed during surgery.

According to the 2015 American College of Gastroenterology (ACG) clinical guidelines, postoperative surveillance should be done yearly with an endoscopic evaluation of the rectum or ileal pouch, with any concerning polyposis being treated through polypectomy or proctectomy [17]. The 2020 American Society of Gastrointestinal Endoscopy (ASGE) guideline recommends pouchoscopy in IPAA patients at one- to two-year intervals [16].

Despite clear guidance by major GI societies, inadequate surveillance remains a major concern. In a study of 150 patients with FAP and their at-risk family members, only 54% of FAP patients and 42% of family members recently completed the recommended colorectal cancer surveillance tests [18]. The following three factors found to be significantly related to inadequate surveillance were patient reports that they had not received a recommendation to have endoscopic colon surveillance, lack of health insurance or of insurance reimbursement for the screening test, and belief that they were at average or reduced risk of colon cancer for their age, sex, or race. In our case, the patient endorsed all three factors playing a role in his lack of follow-up and appropriate surveillance.

Taking those factors into account, the barriers to adequate surveillance in FAP appear to mirror the challenges faced in achieving adequate colorectal cancer screening rates in the general population. These challenges fall mainly under the umbrella of social determinants of health (SDH). In a large review of studies spanning from 1970 to 2019, researchers determined that social support or isolation, poverty, education level, and immigration status played the biggest role in colorectal cancer survival [19]. Furthermore, less social support and lower socioeconomic status were associated with a more advanced colorectal cancer stage at diagnosis. Our patient had social support challenges after the passing of his mother, socioeconomic challenges related to a lack of insurance coverage, and a lack of education sufficient to understand the high risks associated with his condition. Without addressing the SDH, it is likely that more preventable cases of advanced malignancy will be seen in the postoperative FAP patient population.

Considering the SDH in FAP patients and the significant gap between surveillance guidelines and actual surveillance, it is clear that more should be done to avoid these unfortunate outcomes. In a study of 236 FAP patients in the Finnish Polyposis registry, patients who were called to set up screening had much earlier interventions and significantly longer life expectancy compared with patients who were not diagnosed until symptoms appeared [20]. This lends evidence to how small interventions can lead to improved surveillance and outcomes. Improved access to a primary care physician could provide more opportunities to improve surveillance rates or detect concerning findings earlier through history and physical examination (including digital rectal examination). Given the challenges associated with polyposis syndromes, this case presents several opportunities to expand current management and guidelines for patients with FAP. The surgical approach can be individualized based on both the patient’s clinical situation and a detailed assessment of socioeconomic risk factors. Social determinants of health can be further addressed through the expansion of patient education and support resources to improve guideline-based postoperative surveillance.

## Conclusions

We present a rare case of a young male with FAP presenting 20 years after restorative proctocolectomy with IPAA, without interval surveillance examinations, found to have metastatic rectal adenocarcinoma. Even in the setting of proctocolectomy, there is often a rectal cuff still present, which further highlights the importance of both ACG and ASGE recommending close surveillance examinations. Despite this, follow-up is frequently inadequate. Hence, consideration of individualized surgery, such as cuffless IPAA, can help further minimize the risk of potential malignancy in high-risk patients like ours. Additionally, this case reinforces the importance of considering social determinants of health, including patient education and support resources, in future guidelines as a way to improve surveillance needed in patients with polyposis syndromes.
